# Identification of Loci Affecting Accumulation of Secondary Metabolites in Tomato Fruit of a *Solanum lycopersicum* × *Solanum chmielewskii* Introgression Line Population

**DOI:** 10.3389/fpls.2016.01428

**Published:** 2016-09-28

**Authors:** Ana-Rosa Ballester, Yury Tikunov, Jos Molthoff, Silvana Grandillo, Marcela Viquez-Zamora, Ric de Vos, Ruud A. de Maagd, Sjaak van Heusden, Arnaud G. Bovy

**Affiliations:** ^1^Wageningen University and Research CentreWageningen, Netherlands; ^2^Institute of Biosciences and Bioresources, National Research Council of ItalyPortici, Italy; ^3^Centre for Biosystems GenomicsWageningen, Netherlands

**Keywords:** tomato (*Solanum lycopersicum*), QTL analysis, flavonoids, alkaloids, introgression lines

## Abstract

Semi-polar metabolites such as flavonoids, phenolic acids, and alkaloids are very important health-related compounds in tomato. As a first step to identify genes responsible for the synthesis of semi-polar metabolites, quantitative trait loci (QTLs) that influence the semi-polar metabolite content in red-ripe tomato fruit were identified, by characterizing fruits of a population of introgression lines (ILs) derived from a cross between the cultivated tomato *Solanum lycopersicum* and the wild species *Solanum chmielewskii*. By analyzing fruits of plants grown at two different locations, we were able to identify robust metabolite QTLs for changes in phenylpropanoid glycoconjugation on chromosome 9, for accumulation of flavonol glycosides on chromosome 5, and for alkaloids on chromosome 7. To further characterize the QTLs we used a combination of genome sequencing, transcriptomics and targeted metabolomics to identify candidate key genes underlying the observed metabolic variation.

## Introduction

Tomato (*Solanum lycopersicum*) is one of the most important vegetable crops worldwide with more than 160 million tons produced in 2013 (FAOSTAT, 2015)^1^. This crop has served as a model organism for fleshy fruit plants and the complete genome sequence of one reference genome and up to 500 re-sequenced accessions is now available through the Solanaceae Genome Network (SGN)^2^ (Tomato Genome Consortium, 2012). As with many other crop plants, tomato has been subjected to intensive domestication and breeding activities, which reduced the genetic variability in commercial materials. Domestication has been focused on yield, disease resistance, color and shape, while taste and nutritional value have long been neglected (Lin et al., 2014). Currently, there is a growing demand to introduce novel genetic variation in commercial tomato in order to improve quality traits such as flavor and nutritional value. This genetic variation can be found in mutagenized populations, in core collections and in wild species and in introgression lines (ILs) derived from those. The potential of wild species as sources for genetic improvement of crops is increasingly recognized. A major goal of modern tomato breeding is to screen crossable wild *Solanum* species, such as *Solanum lycopersicoides*, *Solanum pennelli*, *Solanum habrochaites*, *Solanum chmielewskii*, *Solanum pimpinellifolium*, *Solanum neorickii*, *Solanum peruvianum*, and *Solanum cheesmanii* for valuable traits, such as resistance against various biotic and abiotic stresses (Légnani et al., 1996; Frankel et al., 2003), primary metabolites (Schauer et al., 2005) and secondary metabolites (Alseekh et al., 2015). Wild species have been used as a source to develop ILs in *S. lycopersicum*, resulting in a set of lines each carrying a single or a few well-defined chromosome segments from the exotic germplasm source. These populations can be used to identify quantitative trait loci (QTLs) that improve crop quality once introgressed into an elite genetic background (Zamir, 2001). In addition to desirable traits, wild species also carry many agriculturally undesirable traits. Molecular genetic studies can identify the genetic and physical position of the underlying QTLs and introgression breeding can transfer the desirable traits into commercial varieties, while selecting against the undesirable ones.

The quality of tomato, in terms of nutritional value, taste, fragrance and appearance is essentially determined by its biochemical composition. To improve the quality of the crop, currently much research is devoted to the elucidation of the pathways and mechanisms that lead to the synthesis and accumulation of quality-related metabolites. The identification of QTLs that influence the chemical composition of ripe fruit, by screening IL populations, is an effective first step toward the identification of the underlying key genes that influence the nutritional quality of tomatoes. One of the best examples of this approach is the use of the founder tomato IL population, derived from a cross between the cultivated *S. lycopersicum* cv M82 and the green fruited wild species *S. pennellii* LA0716 (Eshed and Zamir, 1995). This population has been used to identify QTLs for primary metabolites, volatile compounds, as well as semi-polar secondary metabolites, such as flavonoids and alkaloids (Schauer et al., 2006; Semel et al., 2006; Tieman et al., 2006; Mathieu et al., 2009; Toubiana et al., 2012; Alseekh et al., 2015). These analyses also led to the identification of candidate genes involved in specific QTLs (Fridman et al., 2001; Causse et al., 2004; Stevens et al., 2007; Bermudez et al., 2008), some of which have been shown to be the key gene underlying a specific QTL by reverse genetics studies (Zanor et al., 2009), while for others this still remains to be demonstrated. Despite the large number of studies related to primary metabolites and yield-associated traits, far less is known about QTLs determining secondary metabolites, such as flavonoids and alkaloids.

Flavonoids represent a large family of low molecular weight polyphenolic secondary metabolites that are widespread over the plant kingdom. To date, more than 6000 different flavonoids have been described and the number is still growing (Koes et al., 1994). Based on their aglycone structure they can be grouped into several classes, such as chalcones, flavanones, flavonols, anthocyanins, and others. Flavonoids are involved in a diverse range of biological processes, such as pigmentation to attract pollinators and seed dispersers, protection against damage from ultraviolet light and pathogen resistance. In addition, they are associated with human health-promoting properties (Harborne and Williams, 2000; Tapas et al., 2008). In tomato fruits, accumulation of flavonoids is restricted to the peel (Bovy et al., 2002, 2010; Schijlen et al., 2008). The main flavonoids present in tomato fruit peel are the chalcone naringenin-chalcone and various sugar conjugates of the flavonols quercetin and kaempferol. The structural information available about flavonoids and other semi-polar metabolites present in tomato increased substantially in the past decade, thanks to advances made in metabolomics tools, such as liquid chromatography and mass spectrometry (Moco et al., 2006, 2007; Iijima et al., 2008; Mintz-Oron et al., 2008). However, our understanding of the genetic network regulating the accumulation of these compounds in tomato fruit is still incomplete. As indicated above, QTL analyses in interspecific IL populations can be used as a tool to identify key genes of this network in two ways: (i) qualitative and quantitative variation within and between metabolites, established by metabolic profiling of the complete set of ILs, can be used to determine the functional nature of the underlying key genes and (ii) precise knowledge of map positions of introgressions and the tomato genome sequence could facilitate the molecular cloning of these candidate genes. Previously, we demonstrated the success of this approach, by using an IL population derived from a cross between the commercial tomato cultivar *S. lycopersicum* cv. Moneyberg and the wild species *S. chmielewskii* (accession LA1840) to unravel the molecular and biochemical basis underlying the *y* mutation in tomato, which leads to pink-colored tomato fruits (Ballester et al., 2010).

Alkaloids are generally considered as anti-nutritional factors in our diet. Their biological effects in humans range from highly toxic, such as α-solanine and α-chaconine in potato tubers, to bitter tasting, such as α-tomatine in tomato. Domestication and breeding efforts have focused on reducing the levels of these anti-nutrients, but the success has been limited and some of these substances still remain in our daily diet (Friedman, 2002, 2006). In recent years, significant progress has been made in the elucidation of the steroidal glycoalkaloid pathway in Solanaceous species (Iijima et al., 2008, 2013; Mintz-Oron et al., 2008; Itkin et al., 2011, 2013; Cárdenas et al., 2016). In fruit of the cultivated tomato, the bitter tasting α-tomatine is present at high levels in early developmental stages and its levels decrease upon ripening due to its conversion into the acetyl glucosylated forms lycoperoside G, F or esculeoside A, which are not bitter. Putative intermediates in this conversion are hydroxytomatine (also called lycoperoside H), lycoperoside A, B, or C and hydroxylycoperoside A, B, or C, resulting from subsequent hydroxylation, acetylation and a second hydroxylation reactions. Fruits of many wild tomato species accumulate mostly the early, bitter, type of alkaloids (Iijima et al., 2013).

In the current study, we used the *S. chmielewskii* IL population to identify genomic regions controlling the production of semi-polar secondary metabolites, such as alkaloids, flavonoids and other phenylpropanoids, in tomato fruit. By combining biochemical pathway knowledge and genomic information, several candidate genes were identified. Further analysis of a major QTL on chromosome 5 for flavonols revealed the flavonoid pathway gene *chalcone isomerase 1* (*CHI1*) as the key gene underlying the variation in quercetin- and kaempferol glycosides.

## Materials and Methods

### Plant Material and Growth Conditions

The IL population is composed of 34 indeterminate lines containing single or multiple introgressions from the wild species *Solanum chmielewskii* (LA1840) in the background of the commercial tomato variety *Solanum lycopersicum* cv. Moneyberg. The 34 ILs were grown in two greenhouses located in Avignon (Southern France) and Wageningen (The Netherlands) during spring and summer of 2007. From them, 25 were grown in both locations, five only in Avignon and four only in Wageningen. The day/night temperature set points were 25/15°C and 21/19°C in Avignon and Wageningen, respectively. At least nine plants were grown per each IL and each biological replicate consisted of at least six ripe fruit obtained from three different plants. Whole fruit was sampled from the plants grown in Wageningen, while fruit pericarp from plants grown in Avignon. After harvesting and sampling, the fruit material was immediately frozen in liquid nitrogen, ground to a fine frozen powder using an analytical electric mill and stored at -80°C until used for further analyses.

Selected ILs were grown again during the spring and summer of 2008 in Wageningen (The Netherlands). Samples were harvested at four stages of ripening (mature green (G), breaker (B), turning (T), and red (R)), which were judged by the fruit appearance and firmness. Subsequently, the fruit peel was carefully separated from the rest of the fruit (the flesh tissue) using a scalpel. Both flesh and peel tissues were immediately frozen in liquid nitrogen and stored at -80°C until used. Each biological replicate consisted of at least six fruit of the same ripening stage obtained from two different plants.

### IL Genotyping

Illumina^®^ infinium bead array was used for a high resolution mapping of the IL population. This analysis was performed as described in Viquez-Zamora et al. (2013) according to the Illumina^®^ Infinium^®^ HD Assay protocol: (Illumina^®^ Infinium^®^ HD Assay Ultra Protocol Guide. California, USA: ^©^Illumina, Inc; 2009. pp. 1–224. Catalog #WG-901-4007). The complete information of the SNP markers used is available at http://www.plantbreeding.wur.nl/Publications/SNP/4072SNP-Sequences.xlsx.

In addition, a set of PCR-based markers consisting of 130 COSII markers (Wu et al., 2006) and three simple sequence repeats (Frary et al., 2005), previously mapped in the tomato genome and covering all 12 tomato chromosomes, were used to genotype the *S. chmielewskii* IL population (Prudent et al., 2009; Do et al., 2010). Sequences of the primers are available on the Solanaceae Genomics Network Web site.

For the COSII markers, amplicon size differences between the two parents were detected in 12% of the cases and were used to genotype the IL population directly; in the other cases, the amplicons were digested with different restriction enzymes (*TaqI*, *HinfI*, *AluI*, *DraI*, *RsaI*, and *MseI*) to identify polymorphisms. Where no polymorphisms were detected, single-band amplicons were purified and sequenced. Amplicon sequences were aligned and examined for polymorphisms using the program CAPSdesigner^3^. Thereafter, the IL population was genotyped via cleaved-amplified polymorphic sequence assays (Konieczny and Ausubel, 1993).

### Analysis of Semi-polar Metabolites by LC-PDA-QTOF-MS

Semi-polar metabolites were extracted according to Moco et al. (2006). Briefly, 500 mg of fresh weight tissue were extracted with 1.5 mL pure methanol (final methanol concentration in the extract approximately 75%). The samples were sonicated for 15 min, filtered through 0.2 μm inorganic membrane filter and 5 μL were used for the analysis.

Liquid chromatography quadrupole time of flight-mass spectrometry analyses (LC-PDA-QTOF-MS) were carried out according to Bino et al. (2005). The detected flavonoid compounds were identified using authentic standards and accurate mass liquid chromatography mass spectrometry analysis using public databases (Moco et al., 2006; Iijima et al., 2008).

### RNA Isolation and qRT-PCR Gene Expression Analysis

Total RNA was isolated from 150 mg of tomato fruit tissue using 1.50 mL of Trizol reagent (Invitrogen) according to the manufacturer’s instructions. Before cDNA synthesis, total RNA was treated with DNase-I Amplification Grade (Invitrogen) and purified with an RNeasy Mini Kit (Qiagen). And aliquot of 1 μg of total RNA was used for cDNA synthesis using the iScript cDNA synthesis kit (Bio-Rad Laboratories) in a 20-μL final volume according to the manufacturer. Expression levels of each gene were measured in duplicate reactions, performed with the same cDNA pool, in the presence of fluorescent dye (iQ SYBR Green Supermix) using an iCycler iQ instrument (Bio-Rad Laboratories) with specific primer pairs (Supplementary Table 5) (Ballester et al., 2010). The constitutively expressed mRNA encoding ubiquitine was used as internal reference. Expression levels were determined relative to the internal reference and multiplied by a factor 10. Calculations of each sample were carried out according to the comparative Ct method.

### Microarray Analysis

The transcript profiling analysis was done using whole fruit tissue. Three biological replicates – pools of at least six fruit per plant were analyzed. Total RNA was extracted as described for real-time quantitative PCR. The 100 ng of total RNA was used to synthesize cDNA using Ambion WT expression kit (Applied Biosystems/Life Technologies, Nieuwekerk a/d IJssel, The Netherlands), which was subsequently labeled with biotin using the Affymetrix GeneChip WT Terminal Labeling Kit (Affymetrix, Santa Clara, CA, USA) and hybridized to Affymetrix EUTOM3 tomato exon arrays (Affymetrix). The microarray signals were determined using MadMax microarray analysis software^4^. The raw data can be found in Supplementary Data Sheet 1. Further analysis was performed using Genemaths XT microarray data analysis software (Applied Maths)^5^. Prior to analysis, the data were normalized using 2log transformation and subsequently scaled by subtraction of the mean (for each compound over the samples).

Student’s *t*-test was performed in Genemath XT, the Pearson correlation coefficients were calculated using the corresponding function of Microsoft Office Excel 2010.

### Cloning, Sequencing, and Mapping of *CHI1* Gene

Full-length cDNA sequences were amplified from ripe whole fruit of cv. Moneyberg, IL5b and IL7d using the SMART RACE cDNA amplification kit (Clontech Laboratories). Genomic DNA was amplified from leaves of the parental lines *S. lycopersicum* cv. Moneyberg and *S. chmielewskii* LA1840 using the GenomeWalker kit (Clontech Laboratories). The amplified sequences of both full-length cDNAs and genomic DNAs were cloned into pGEM-T-Easy vector (Promega) and sequenced.

## Results

### Physical Mapping of the *S. lycopersicum × S. chmielewskii* IL Population

**FIGURE 1:**
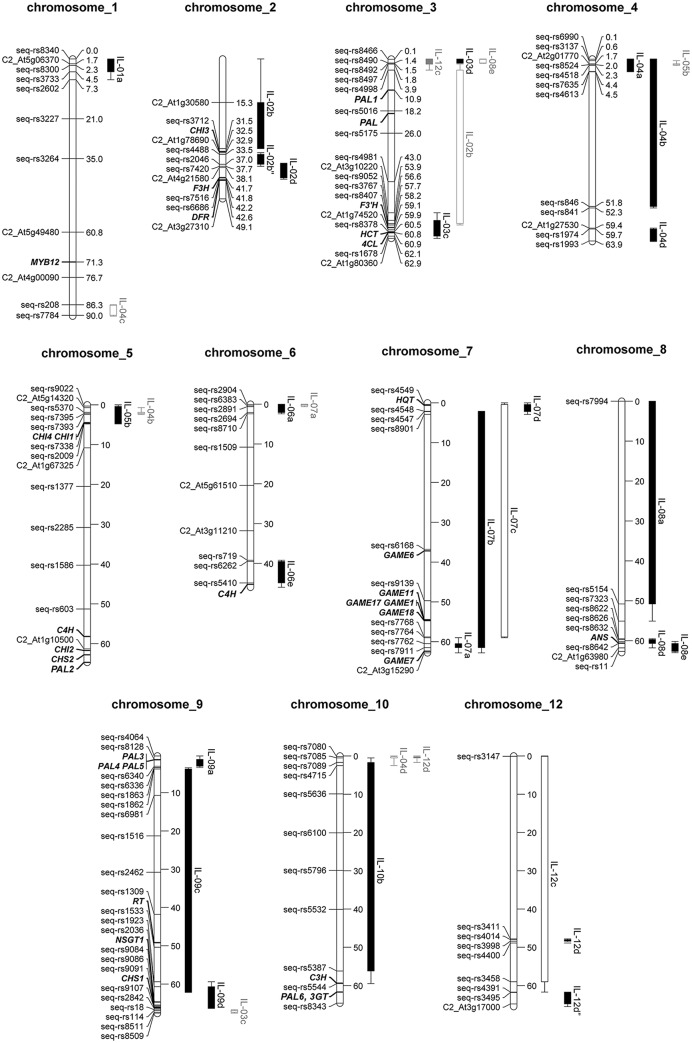
**Physical map of the subset of *S. lycopersicum* x *S. chmielewskii* introgression lines used in this study.** Markers flanking introgressions and candidate genes are shown on the left side of chromosomes and their physical positions (in Mbp) – on the right side. Homozygous and heterozygous introgressions are depicted by filled and empty rectangles, respectively, on the right side of chromosomes. Whiskers indicate a distance between *S. chmielewskii* and *S. lycopersicum* alleles of markers flanking introgressions, therefore showing how far introgressions could possibly stretch. Introgressions depicted in gray are minor introgressions, which are detected in an IL carrying a major introgression in a different chromosome. For precise genomic marker positions see Supplementary Table 1.

The objective of this study was to discover genetic and genomic regions of *S. chmielewskii* LA1840 that affect accumulation of secondary metabolites in fruits of the commercial tomato *S. lycopersicum* cv. Moneyberg, as a first step toward discovering genes acting in the related metabolic pathways. For this purpose we analyzed a population of 25 *S. chmielewskii* LA1840 ILs. For 20 of them the first linkage maps, based on COSII and SSR markers, were presented in Prudent et al. (2009) and Do et al. (2010). According to these data, 14 ILs each carried a single wild chromosomal introgression. In our study a high resolution genome wide SNP array, consisting of 5,528 SNP markers (Viquez-Zamora et al., 2013) was used to determine physical boundaries of the ILs. 1,660 markers were found to be polymorphic between *S. chmielewskii* LA1840 and *S. lycopersicum* cv. Moneyberg. As a result, out of the 25 ILs analyzed, 15 ILs were found to carry a single, homozygous *S. chmielewskii* introgression and 10 ILs carried two or more introgressions in one or in multiple chromosomes (**Figure 1**; Supplementary Table 1). Four major heterozygous introgressions were found on chromosomes 1, 3, 7, and 12.

### Metabolic Profiling of the *S. chmielewskii* ILs Using Liquid Chromatography Coupled to Mass Spectrometry (LC-MS)

The *S. chmielewskii* IL population was grown at two different locations, Wageningen (The Netherlands) and Avignon (France), and ripe fruits were harvested. Three biological replicates were created by pooling fruit material of three independent plants per replicate. Semi-polar metabolites of ripe fruits were profiled using LC-MS. A total of 126 compounds were putatively identified in tomato fruit based on public mass spectral databases (Moco et al., 2006; Iijima et al., 2008). The mass spectra and the retention times were compared with authentic chemical standards when available (Supplementary Table 2). Different biochemical families of secondary metabolites were identified, including alkaloids, flavonoids (flavanones, flavones, and flavonols) and other phenylpropanoids.

**FIGURE 2:**
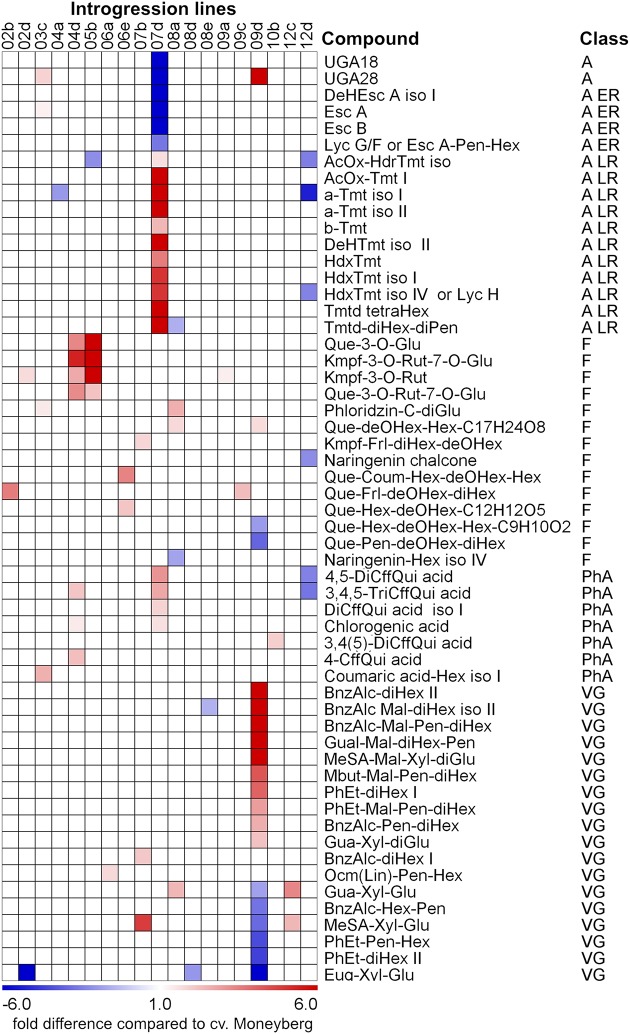
**Heat map of fold differences in accumulation of non-volatile secondary metabolites between ripe fruits of the *S. chmielewskii* ILs compared to fruits of the recipient parent *S. lycopersicum* cv. Moneyberg.** The differences represented in the heat map were significant (*p* < 0.05) in both the geographical locations where fruits of the ILs were harvested. Compound classes: A – alkaloids; A ER – alkaloids, which are present at early ripening stage of *S. lycopersicum* fruits; A LR - alkaloids, which are present at a later ripening stage of *S. lycopersicum* fruits; F – flavonoids; PhA – phenolic acids; VG – glycosylated volatile compounds. For the exact ratio values see Supplementary Table 3.

Analysis of variance (ANOVA) showed that the content of 56 compounds was significantly affected (*p* < 0.05) in fruits of the ILs compared to fruits of cv. Moneyberg in both growing locations (**Figure 2**; Supplementary Table 3). Glycosylated volatile organic compounds (VOCs), alkaloids, and flavonoids were the most representative – 18, 17, and 14 compounds, respectively. Introgression in chromosomes 9 (IL9d) and 7 (IL7d) appeared to have the largest effects on the accumulation of different types of glycosylated VOCs and alkaloids, respectively. Two introgressions, on chromosomes 4 (IL4d) and 5 (IL5b), had a major effect on the accumulation of tomato fruit flavonols.

### Introgression 5b in Chromosome 5 Increases the Accumulation of Kaempferol and Quercetin Glycosides

**FIGURE 3:**
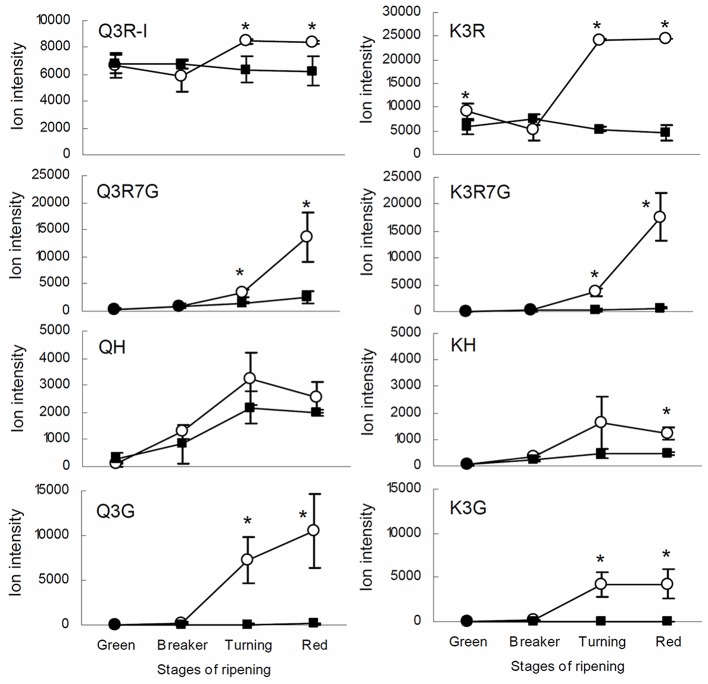
**Flavonol glycosides in tomato fruit.** Ion intensity of the quercetin and kaempferol glycosides in the peel of tomato fruit of IL5b (white circles) and cv. Moneyberg (black squares) during four stages of ripening: green, breaker, turning and red. Values are the average of three biological replicates, including the standard deviation. Stars (^∗^) indicate significant changes based on a *t*-test (*p* < 0.05).

**FIGURE 4:**
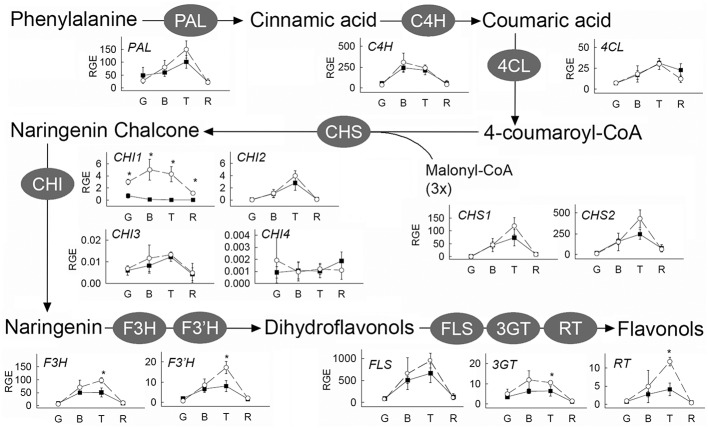
**Relative expression of phenylpropanoid and flavonoid genes in peel of cv. Moneyberg (black squares) and IL5b (white circles) tomatoes at different stages of ripening (Mature green (G), Breaker (B), Turning (T), Red (R)).** Expression levels were determined by qRT-PCR and expressed relative to the expression of the ubiquitin gene. Values represent averages of three biological replicates, each with two technical replicates. Stars (^∗^) indicate significant changes based on a *t*-test (*p* < 0.01). Identifiers (Solyc numbers) and primers of the genes tested are shown in Supplementary Table 5.

The result from the IL screening showed that the largest quantitative changes in levels of flavonols in ripe fruits were due to the presence of the IL5b introgression (**Figure 2**; Supplementary Table 3). To further investigate the accumulation of kaempferol and quercetin glycosides in tomato fruit, metabolic profiling was performed in different ripening stages (mature green (G), breaker (B), turning (T), and ripe (R)) of IL5b fruits and the control cv. Moneyberg. Since flavonoids normally accumulate in the fruit peel only (Muir et al., 2001; Bovy et al., 2002; Colliver et al., 2002), we decided to focus the LC-MS analyses on methanolic extracts of the peel of IL5b and cv. Moneyberg fruits to increase the sensitivity of the measurements. An increase in the flavonols quercetin- and kaempferol-3-*O*-rutinoside (denoted as Q3R and K3R, respectively), quercetin- and kaempferol-3-*O*-rutinoside-7-*O*-glucoside (Q3R7G and K3R7G) and quercetin/kaempferol-3-*O*-glucose (Q3G and K3G) was observed in the fruit peel of IL5b compared to cv. Moneyberg, although the extent of the differences appeared to be compound-dependent (**Figure 3**). In order to unravel the genetic factors associated with the different patterns of accumulation of quercetin and kaempferol glycosides caused by the introgression in IL5b, a transcriptomics analysis of the ripening fruits (G, B/T, and R) was performed using the EU-TOM3 Affimetrix microarray. A total of 511 genes predicted by the International Tomato Annotation Group (ITAG) (Tomato Genome Consortium, 2012) were located in the chromosomal region corresponding to the IL5b introgression. Expression levels of 17 genes in the IL5b introgression region were found to be upregulated threefold or higher in turning fruits of IL5b compared to turning fruits of cv. Moneyberg (Supplementary Table 4). Among them, one gene – *CHALCONE ISOMERASE 1* (*CHI1*) (Solyc05g010320) is directly involved in the flavonoid biosynthesis pathway (Bovy et al., 2002). To corroborate the results from the microarray experiments, the expression of a set of known fruit-expressed biosynthetic genes involved in the phenylpropanoid/flavonoid pathway (Ballester et al., 2010) (Supplementary Table 5) was analyzed also in ripening fruit peel samples of IL5b and cv. Moneyberg, using qRT-PCR (**Figure 4**). Furthermore, we tested the expression of three additional putative *CHI* genes, which we denoted as *CHI2* (Solyc05g052240), *CHI3* (Solyc02g067870, BQ505699), and *CHI4* (Solyc05g010310). Most of the genes tested showed a similar ripening-correlated pattern of expression in both IL5b and cv. Moneyberg: expression levels of phenylalanine ammonia-lyase (*PAL*), coumaroyl-4-hydroxylase (*C4H*), 4-coumarate ligase (*4CL*), chalcone synthases (*CHS1* and *CHS2*), chalcone isomerases 2 and 3 (*CHI2* and *CHI3*), flavonoid-3-hydroxylase (*F3H*), flavonoid-3′-hydroxylase (*F3′H*), flavonol synthase (*FLS*), flavonoid-3-*O*-glucosyltransferase (*3GT*) and flavonoid 3-*O*-glucoside-rhamnosyltransferase (*RT*) increased during ripening, peaked at B/T stage and decreased in ripe fruits. In contrast, the ripening-regulated pattern of *CHI1* expression in cv. Moneyberg was opposite to the other genes in the phenylpropanoid/flavonoid pathway. This gene showed a low expression at G stage, which decreased even more at the later stages of ripening. This confirmed earlier observations (Muir et al., 2001; Bovy et al., 2002) that low expression of *CHI1* is a major bottleneck in the biosynthesis of flavonols in fruits of cultivated tomatoes, such as cv. Moneyberg. In line with the microarray results, *CHI1* expression in IL5b was significantly increased compared to cv. Moneyberg at G, B and T stages, suggesting that *CHI1* expression relieves the block of the pathway in fruits of IL5b, which makes it the primary candidate gene for the flavonoid QTL mapped on IL5b.

Although all other flavonoid pathway genes did not show such a dramatic difference in expression as *CHI1*, in general, they tended to be expressed at higher levels in breaker and/or turning fruits of IL5b compared to cv. Moneyberg. This suggests that differences in flavonoid content between IL5b and cv. Moneyberg might also be due to coordinate control of flavonoid gene expression during ripening. The MYB12 transcription factor has previously been shown to regulate flavonol biosynthesis in tomato fruit (Adato et al., 2009; Ballester et al., 2010). Another MYB family transcription factor (Solyc05g009720) was found among the genes up-regulated in fruits of IL5b and physically located in the introgression region – at 3.93 Mb on chromosome 5. However, no significant correlation of expression was observed between this *MYB* gene and the 14 biosynthetic genes involved in the phenylpropanoid/flavonoid pathway present on the microarray (Supplementary Table 6). Analysis of two near-isogenic tomato lines only differing for a *S. chmielewskii* introgression in chromosome 5 that starts downstream of the *MYB* gene, but covers the *CHI1* gene, confirmed the high-flavonoid fruit phenotype caused by the presence of the *S. chmielewskii* introgression (results not shown). This supports our conclusion that *CHI1* is the primary candidate gene underlying the flavonoid QTL on chromosome 5.

**FIGURE 5:**
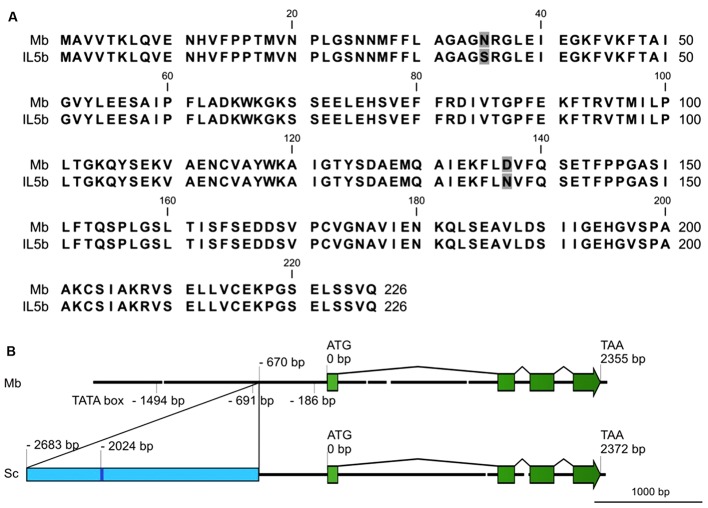
**(A)** Alignment of deduced amino acid sequences of *CHI1* alleles, including the sequences of *S. lycopersicum* cv. Moneyberg (Mb) and the *S. chmielewskii* introgressed line 5b (IL5b). Different amino acids between the sequences are boxed in gray. **(B)** Schematic overview of the genomic structures of the *CHI1* gene in Moneyberg (Mb) and *S. chmielewskii* (Sc). The *CHI1* gene consists of four exons (boxes in green) and three introns. The blue box represents part of ToRTL1, a Ty1/Copia long terminal repeat (LTR) retroelement observed in the promoter region of *S. chmielewskii CHI1* gene.

Full length cDNAs of *CHI1* (Solyc05g010320) were isolated from ripe fruit of both cv. Moneyberg and IL5b using Rapid Amplification of cDNA Ends (RACE). The derived protein sequences differ at only 2 amino acid positions (N35S and D137N in cv. Moneyberg→IL5b, **Figure 5A**). We cannot exclude that these two amino acid differences affect the function of the protein, but consider it unlikely that they account for the changes seen in *CHI1* gene expression.

A genome walking approach was used to analyze and compare the genomic structure of *CHI1* in the parental lines cv. Moneyberg and *S. chmielewskii*. The genomic sequence between both cv. Moneyberg and *S. chmielewskii* showed the presence of three introns, with the highest sequence variation observed in the first intron (**Figure 5B**). Deletions of 16, 38, 14, and 17 bp and an insertion of 17 bp were observed in cv. Moneyberg compared to *S. chmielewskii*. Comparison of the promoter regions, using the TSSP/Prediction of PLANT Promoters tool at the RegSite Plant DB (Softberry Inc.), revealed an insertion in the promoter region of *S. chmielewskii* at – 670 bp of the transcription start site, with a size of at least 2,063 bp. The insertion sequence was searched for homology to transposon-like sequences in RepBase (Jurka et al., 2005). In this insertion several fragments were found with 68 to 91% similarity to (from 5′to 3′) (i) twice a Copia-38_ST Long Terminal Repeat fragment from potato, (ii) a 34 nt sequence with high similarity (91%) to the polypurine tract containing region of ToRTL1, (iii) a *Ty1/Copia* long terminal repeat (LTR) retro-element (Daraselia et al., 1996), and (iv) to 3′and 5′ sequences, respectively, of a hAT-like DNA transposon from potato (Jurka and Kohany, 2006).

### IL7d Affects Accumulation of Alkaloids in Tomato Fruit

**FIGURE 6:**
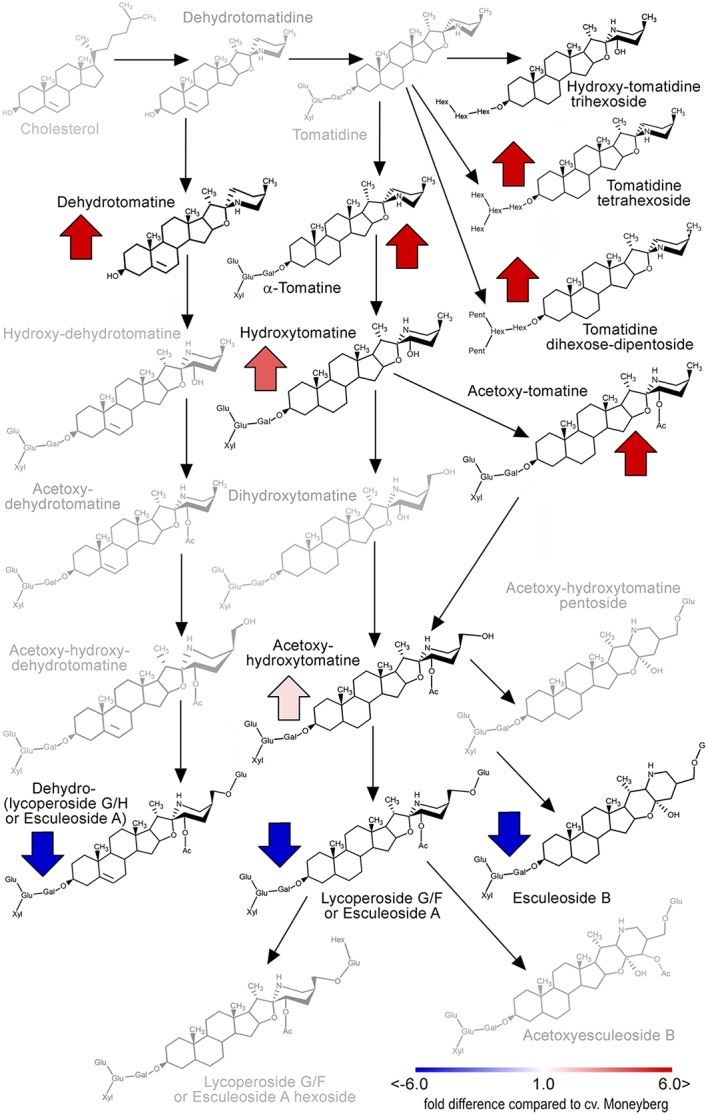
**Alterations in the tomato fruit alkaloid pathway caused by the IL7d introgression (adapted from Itkin et al., 2011).** The intensity of red and blue arrows indicate the extent of quantitative changes of alkaloids compared to their amounts in fruit of cv. Moneyberg. Gray colored compounds were not detected in this study.

According to the marker data IL7d carried a *S. chmielewskii* introgression in the top of chromosome 7 (0–2.86 Mb) (**Figure 1**; Supplementary Table 1). This introgression affected accumulation of two groups of alkaloids in fruits of this IL (**Figure 2**; Supplementary Table 3). α-tomatine, hydroxytomatine (lycoperoside H) and lycoperoside A/B/C had higher levels in fruits of IL7d compared to the control cv. Moneyberg (**Figure 6**), whereas the amounts of esculeoside A and lycoperoside F/G in fruits of this introgression line were reduced by up to 45-fold (**Figure 6**). According to the proposed tomato alkaloid biosynthetic pathway (Mintz-Oron et al., 2008; Itkin et al., 2013) α-tomatine undergoes a number of ripening-induced hydroxylation and glycosylation modifications to produce the esculeoside type glycoalkaloids. Therefore, the accumulation of the green fruit-type alkaloids in fruits of IL7d suggests that this genomic region harbors a genetic factor which prevents or blocks the ripening-dependent glycoalkaloid modification (**Figure 6**). Accumulation of putative intermediates in the proposed pathway, such as hydroxy-lycoperoside A, B, or C, which after glycosylation produce the esculeoside type alkaloids, was not observed in fruits of IL7d. This suggests that the pathway is most likely interrupted at the step of hydroxylation of acetoxytomatine. Hydroxylation of alkaloids and other secondary metabolites is often mediated by enzymes of the cytochrome P450 family.

Three P450s were found to be located in the IL7d introgression region: Solyc07g006140, Solyc07g006890, and Solyc07g007460. Of these three genes, Solyc07g006890 was the most highly expressed in fruits and its transcript level increased during ripening. In ripe fruits of IL7d this gene showed a moderate threefold decrease in expression compared to its average expression observed in fruits of cv. Moneyberg and of IL5b (Supplementary Table 7).

### IL9d Affects the Accumulation of Volatile Glycosides

The introgression 9d in chromosome 9 affected the accumulation of different glycoconjugate forms of volatile compounds, such as guaiacol, methyl salicylate (MeSA), and eugenol (**Figure 2**; Supplementary Table 3). IL9d led to the conversion of xylosyl-glucopyranoside forms of these volatiles into the corresponding xylosyl-diglucopyranoside forms. This conversion was shown to be mediated by the Non-Smoky Glycosyltransferase 1 gene (*NSGT1*), located within the IL9d introgression (**Figure 1**) (Tikunov et al., 2013) and suggests that *S. chmielewskii* carries a functional version of *NSGT1*. Indeed, re-sequencing of the *S. chmielewskii* LA1840 genome using a next generation sequencing approach revealed a gene with 96% homology to *NSGT1* (Supplementary Image 1). In addition to glycosides of guaiacol, MeSA and eugenol, our data revealed that diglycosides of the aroma volatiles benzyl alcohol, 2-phenylethanol, and 2- or 3-methylbutanol were modified in the same manner (**Figure 2**; Supplementary Table 3).

## Discussion

Many metabolite QTLs (mQTLs) have been described in ILs derived from the wild tomato relatives *S. pennellii* or *S. habrochaites* (Schauer et al., 2006; Tieman et al., 2006; Mathieu et al., 2009; Steinhauser et al., 2011; Toubiana et al., 2012; Alseekh et al., 2015). *S. chmielewskii* is another wild species crossable with cultivated tomato, with smaller, green fruits. The influence of the fruit load on the accumulation of dry matter and sugars in tomato fruit and on primary metabolites have been described recently in tomatoes derived from the *S. chmielewskii* IL population (Prudent et al., 2009; Do et al., 2010). In this paper, we used a combination of genomic and metabolomics approaches to identify mQTLs and candidate genes controlling the synthesis of semi-polar compounds in tomato fruit. For this, ripe fruits harvested from the *S. chmielewskii* IL population were analyzed for variation in semi-polar secondary metabolites, using LC-PDA-QTOF-MS. By growing the plants at two different locations we could select for robust mQTLs. The screening revealed quantitative and qualitative changes in metabolites accumulating in specific ILs. The major mQTLs were found in ILs 4d, 5b, 7d, and 9d (**Figure 2**).

### Accumulation of Specific Flavonol Glycosides in Tomato Fruit Related to an Increase in *CHI* Gene Expression

Our metabolic analyses revealed a major ripening-dependent increase of several flavonol glycosides in peel of IL5b compared to cv. Moneyberg (**Figure 3**). Absolute quantification of the main flavonols revealed an increase in quercetin-3-*O*-rutinoside from 30 to 260 mg/kg FW and of kaempferol-3-O-rutinoside from 3 to 35 mg/kg FW in peel of ripe IL5b compared to cv. Moneyberg fruits (Supplementary Image 2). Compared to the levels of these compounds found among a collection of 94 cultivated tomato hybrids (Bovy et al., 2010), the IL9d introgression upgrades Moneyberg tomatoes from a low-flavonol to a high-flavonol round tomato type, with levels comparable to those in cherry tomatoes, which are generally regarded as a much better source of flavonols than round/beef tomatoes.

Chalcone synthase (CHS) is the first enzyme involved in the phenylpropanoid/flavonoid pathway leading to the formation of these semi-polar compounds, most of which are present in a glycosylated form. Most of the biosynthetic genes involved in the flavonoid pathway and also transcription factors involved in the regulation of the biosynthetic genes have been identified (Schijlen et al., 2004; Adato et al., 2009; Ballester et al., 2010) and, due to the availability of the tomato genome sequence (Tomato Genome Consortium, 2012), their physical position and chromosomal location is known (**Figure 1**; Supplementary Table 8). However, due to the complexity of flavonoid modification and the presence of more than 500 different forms of flavonoids in tomato (Moco et al., 2006; Iijima et al., 2008; Grennan, 2009), further analysis is still needed to understand the flavonoid pathway to its full extent.

Most plants do not accumulate chalcones, the first class of flavonoids at the top of the biosynthetic pathway. After its formation, naringenin chalcone is usually rapidly isomerized by chalcone isomerase (CHI) to form the flavanone naringenin, a process that may also occur spontaneously in the absence of active CHI. However, in tomato fruit, low expression of *CHI* is rate-limiting and naringenin chalcone is the predominant yellow pigment that accumulates in the peel (Muir et al., 2001; Bovy et al., 2002). Four different putative *CHI* genes have been annotated in tomato, which share at most 75% identity at the amino acid level. The first one (Solyc05g010320), *CHI1*, has been described by Bovy et al. (2002) and its expression was low in ripe tomato fruit, explaining the accumulation of the CHI substrate naringenin chalcone. *CHI2* (Solyc05g052240) expression and CHI activity was increased in *Del/Ros1* transgenic plants accumulating anthocyanins (GenBank acc. no. ES893795) (Butelli et al., 2008), and the expression of the third one, here called *CHI3* (Solyc02g067870, BQ505699), was up-regulated in transgenic plants overexpressing the MYB transcription factor *ANT1* leading to anthocyanin pigmentation in the fruit (Mathews et al., 2003). The expression of the fourth one (Solyc05g010310, here called *CHI4*), is very low compared to the other *CHI* genes (**Figure 4**). Based on the genome annotation, *CHI1*, *CHI2* and *CHI4* are located on chromosome 5, while *CHI3* is located on chromosome 2 (**Figure 1**).

There are two major arguments supporting the conclusion that *CHI1* is the key gene underlying the flavonoid QTL on chromosome 5. Firstly, after analyzing the expression of the biosynthetic flavonoid genes in tomato, *CHI1* showed the highest expression increase in IL5b compared to cv. Moneyberg and was the only gene whose expression was significantly increased in IL5b compared to cv. Moneyberg at all stages of fruit ripening (**Figure 4**; Supplementary Table 4). The increase of the expression of this gene might redirect the flux of the pathway toward the formation of flavonol glycosides, as shown in the results of semi-polar metabolites detected in the fruits of IL5b and in line with results found in transgenic plants overexpressing the petunia *CHI1* gene (Muir et al., 2001). Secondly, *CHI1* is located within the IL5b introgression and is among the 17 genes (out of 511) mapping in this region with an expression level at least threefold higher in turning IL5b fruits relative to cv. Moneyberg fruits (Supplementary Table 4).

IL5b is not a pure line in the sense that a small (<0.5 Mb) additional introgression region in chromosome 4 was also detected in this IL (**Figure 1**). However, within the subset of the *S. chmielewskii* population analyzed in this study, there are several other ILs with introgressions overlapping with the region in this chromosome. None of these lines showed an increase of flavonol glycosides compared to cv. Moneyberg and therefore we consider it unlikely that genes of this introgressed fragment might be responsible for the IL5b flavonoid QTL. In addition, NILs only differing in a chromosome 5 introgression showed a contrasting flavonoid accumulation pattern (results not shown), supporting that the flavonoid QTL is indeed due to the chromosome 5 introgression.

The lack of *CHI1* gene expression in cultivated tomato might be due to (i) a mutation in a promoter regulatory sequence (*cis*-effect) and/or (ii) a mutation in a transcription factor responsible for expression of *CHI* (Willits et al., 2005) (*trans*-effect). We cannot completely exclude the latter possibility and, in this respect, we found a possible candidate *MYB TF* gene in the IL5b QTL region, which was upregulated in T stage fruit of IL5b. However, the expression pattern of this *MYB* gene during ripening was not correlated with the expression of *CHI1*, nor with the expression of the other flavonoid genes tested, which argues against a causal role for this candidate gene. After analyzing the genomic structure of the *CHI1* gene, our results revealed the presence of several repetitive sequences related to transposons and retrotransposons of tomato and potato. Many examples exist of (retro-)transposon insertions influencing expression of down-stream genes such as, in tomato, retrotransposon ToRTL1 driving high expression of the 3-hydroxy-3-methylglutaryl coenzyme a reductase gene 2 (HMG2) (Daraselia et al., 1996). A possible influence of the upstream transposon-like sequences of *CHI1* on its expression, however, remains to be demonstrated.

### Accumulation of α-Tomatine and Lycoperosides in Tomato Fruit

In our study, fruits of IL7d showed an accumulation of α-tomatine, hydroxytomatine and lycoperoside A, B, or C, while levels of lycoperoside G, F and esculeoside A were low compared with cv. Moneyberg fruit. None of the above-mentioned intermediates could be detected in fruits of IL5b or cv. Moneyberg.

A cluster of alkaloid biosynthesis genes has been previously discovered at the bottom of tomato and potato chromosome 7 (Itkin et al., 2013). Our genetic and metabolic data showed that there might be another genetic factor(s) at the top of this chromosome. The accumulation of the compounds from the first steps of the putative alkaloid pathway could be due to (i) a mutation of a gene involved in the hydroxylation of lycoperoside A, B, or C, or (ii) a mutation in regulatory element, such as a transcription factor (TF), responsible for the expression of the hydroxylation gene(s). An example of TF-mediated regulation of alkaloid biosynthesis has been recently shown by Cárdenas et al. (2016). Cytochrome P450s can catalyze aromatic hydroxylations, aliphatic hydroxylations and skeleton formation in secondary metabolite pathways in plants (Ayabe and Akashi, 2006), and therefore CYP’s would be good candidates for further studies. The evaluation of the genomic region of the IL7d introgression region revealed the presence of three cytochrome P450 genes: Solyc07g006140 (SL2.40ch07: 984510-988395 bp), Solyc07g006890 (SL2.40ch07: 1747021-1748529 bp), and Solyc07g007460 (SL2.40ch07: 2165949-2167526 bp). Further functional analysis of these candidate genes is currently underway.

### Glycosylation of Tomato Fruit Volatiles

Our results indicate that the IL9d introgression carries a functional version of the *NSGT1* gene, which mediates the conversion of xylosyl-glucopyranosides of the phenylpropanoid volatiles guaiacol, methyl salicylate and eugenol into the corresponding xylosyl-diglucopyranosides. This conversion affected the release of the corresponding volatiles and subsequently the fruit aroma (Tikunov et al., 2010, 2013). In addition to glycosides of guaiacol, MeSA and eugenol, the present data showed that glycosides of other volatile compounds which play a role in tomato fruit aroma, such as benzyl alcohol, 2-phenylethanol, and 2- or 3-methylbutanol were modified in the same manner. This suggests that these diglycosides may be used as a substrate of the NSGT1 enzyme as well. This hypothesis could indeed be confirmed by metabolic analysis of transgenic *NSGT1*fruits (Tikunov et al., 2013) (Supplementary Image 3). In contrast to phenylpropanoid volatiles, the changes in glycosylation pattern of the other volatiles did not affect their release, neither in IL9d fruits (Supplementary Image 4), nor in transgenic *NSGT1* fruits (Tikunov et al., 2013). This suggests that, in addition to *NSGT1*-mediated glycosylation of the third sugar, the identity of the first two sugar conjugates and the interaction with putative glycoside hydrolases are important determinants for the release of aroma volatiles. We are currently aiming to get a better understanding of the various aspects of volatile “logistics” in tomato fruit and explore the opportunities to influence tomato fruit aroma by manipulating the volatile glycosylation status and thereby the release of these volatiles.

## Conclusion

We identified a number of mQTLs involved in the production of semi-polar metabolites, by examining an IL population derived from a cross between *S. lycopersicum* cv. Moneyberg and *S. chmielewskii* LA1840. The use of specific *S. chmielewskii* ILs in combination with the knowledge gained on these mQTLs and the underlying candidate genes can be used to breed for tomatoes with improved quality. Reverse genetics is required to further elucidate the function of specific candidate genes, in order to gain a better understanding of the biosynthetic pathways leading to the synthesis and accumulation of health-related compounds.

## Author Contributions

A-RB carried out the research and wrote the manuscript. YT was responsible for the metabolomics analyses. JM was involved in the research, SG was involved in writing and correcting the manuscript, MV-Z and SH were involved in the marker analyses, RM was involved in manuscript preparation and data analysis, AB was involved in supervising the project, data analysis, and writing.

## Conflict of Interest Statement

The authors declare that the research was conducted in the absence of any commercial or financial relationships that could be construed as a potential conflict of interest.

## Footnotes

1 http://faostat3.fao.org/

2 http://solgenomics.net

3 https://solgenomics.net/tools/caps_designer/caps_input.pl

4 https://madmax.bioinformatics.nl

5 http://www.applied-maths.com/genemaths-xt
