# Supplementary material for: Identification of Loci Affecting Accumulation of Secondary Metabolites in Tomato Fruit of a Solanum lycopersicum × Solanum chmielewskii Introgression Line Population
Source: Front Plant Sci. 2016 Sep 28;7:1428. doi: 10.3389/fpls.2016.01428 (PMC5040107; doi:10.3389/fpls.2016.01428)
Supplement: Supplementary file 10 [file Image_1.PDF]

```
NSGT1          1 ATGGAGAGAATTAAGGAAAATAGTCCTAGTATTCTATTATTTCCATGGTTAGGTTTTGGCCATGTAAATCCTTTTTTGGCACTAGCCAAGAAATTATCAAAAATGAATTTTCACATATATTTTCTCTCAACACCAATTAT
NSGT1_Chm      1 ATGGAGAGAATTAAGGAAAATAGTCCTAGTATTCTATTATTTCCATGGTTAGGTTTTGGCCATGTAAATCCCTTTTTGGCACTAGCCAAGAAATTATCAAAAATGAATTTTCACATATATTTTCTTTCAACACCAATTAT

NSGT1        141 TCTCAAATCTATCAAGGAAACCCTAGATAAAAACTCAACTAATTATAATCAATCCATACAACTTGTTGAATTTCACTTGCCTTATTTGCATGAGTTACCACCTCATTACCATACAACTAAAGACCTCCCTCCCCATCTCA
NSGT1_Chm    141 TCTCAAATCCATCAAGAAAACTCTAGATAAATACTCAACTAATTATAATATCTCCATACAACTTGTTGAATTTCACTTGCCTTATTTGCATGAGTTACCACCTCATTACCATACAACTAAAGACCTCCCTCCCCATCTCA

NSGT1        281 ACTCCACTCTTATTCAAGCCTTTCAAATGGCTTCTTCCAAATTTCCAAGCATAATTGAAACCCTAAAACCTAACTTGATTATATATGATGGGTTCCAACCATGGGTAGCAACTATGGCTTCATCATACAATATTCATGCT
NSGT1_Chm    281 ACTCCACTCTTATTCAAGCCTTTCAAATGGCTTCTTCAAAATTTCCAAGCATAATTGAAACCCTAAAACCTAACTTGATTATATATGATGGGTTCCAACCATGGGTAGCAACTATGGCTTCATCATACAATATTCATGCT

NSGT1        421 ATTATGTTTTATGTTTCTTCAACTTCTGGTCTAGCCTACCTTTACCACCAATTTCTTCATGGGAGTTCAAGCCTTACATCTTTTCCATTTTCTTCCATATACCTTCATGACCATGAGATCAAGAAATTAGGCATACAACC
NSGT1_Chm    421 ATTATGTTTTATGTTTCTTCAACTTCTGGTCTTGCCTACATTTACCACCAATTTCTTCATGGGAGTTCAAGCCTTACATCTTTTCCTTTTTCTTCATTGTATCTTCATGACTATGAGATCAAGAAATTAGATATGAAACC

NSGT1        561 AATAAAACCACGCGATGAGAAAGCTTTTGCATACATAATCCTTGAGTCTTTTGAACAATCTCACAACATTGTTTTGTTGAACACTTGTAGGGAGATTGAGGGGAAGTATATAGATTATGTTTCTACAATAGGAAAGAAAG
NSGT1_Chm    561 AATAAAACCACGCGATGAGAAAGCTTTTGCATACATAATCCTTAAGTCTTTTGAACAATCTCACAACATTGTTTTGTTGAACACTTGTAGGGAGATTGAGGGGAAGTATATAGATTATGTTTCTACAATAGGAAAGAAAG

NSGT1        701 AGTTGATACCAATTGGACCATTAATTCGCGAGGCGATGATAGATGAGGAGGAGGATTGGGGGACAATTCAATCTTGGCTAGACAAGAAGGATCAATTATCATGTGTTTATGTATCATTTGGAAGTGAAAGCTTCTTGTCA
NSGT1_Chm    701 AGTTGATACCAATTGGACCACTAATTCGCGAGGCGACCATAGGTGAGGAGGAGGATTGGGGGACAATTCAATCTTGGCTAGACAAGAAGGATCAATTATCATGTGTTTATGTATCATTTGGAAGTGAATACTTCTTGTCA

NSGT1        841 AAGCAAGAAATTGAAGAGATAGCAAAAGGGCTTGAGCTAAGCAAAGTTAATTTTATTTGGACAATCAAATTTCCTAAAGGGGTGAACAAAACAATTGAAGAAATGGTTCCACAAGGTTTTCTTGAAAGTACAAAGGGAAA
NSGT1_Chm    841 AAGCAAGAAATTGAAGAGATAGCAAAAGGTCTTGAGTTAAGTAAAGTTAGCTTTATTTGGACAATCAAATTTCCAAAAGGGGTGAACACAACAATAGAAGAAATGGTTCCACAAGGTTTTCTTGAAAGTACAAAGGGAAA

NSGT1        981 AGGGATGGTTATTGAAGGATGGGCACCACAAAGTCTAATTTTGAACCATTCAAGCATTGGAGGTTTTATAACTCATTGTGGATGGAACTCGATTTTAGAAAGCATGAGTTTTGGCATACCAATAATAGCCATGCCTATGA
NSGT1_Chm    981 AGGGATGGTTATTGAAGGATGGGCACCACAAAGTCTAATTTTGAACCATTCAAGCATTGGAGGTTTTGTAACTCATTGTGGATGGAATTCGATTTTGGAAAGCATGAGTTTTGGTATACCAATAATAGCCATGCCTATGA

NSGT1       1121 ATCATGATCAACCATTGAATTCAAGATTAGTGGAGGAACTTGGCATAGGGGTGGAGATTTTGAGAGGTGAAAATGGGAAAATAATGAAAGAAGAGGTGGCAAAAGGAATAAGGAAAGTGATAGAGGAGAAGCCTAGGAAA
NSGT1_Chm   1121 ATCATGATCAACCATTGAATTCAAGATTAGTGGAGGAACTTGGCATAGGGGTGGAGATTTTGAGAGGTGAAAATGGGAAAATAATGAAAGAAGAGGTGGCAAAAGGAATAAAGAAGGTGGTAGAGGATAACACTAGGAAA

NSGT1       1261 CAAATTCATTTGAAGGCAATGCAATTGAGTGAGAAGATAAAATTGAAAGCTATTGATGAAGGGGTTAAAAAGTTGTTGAAGCTATTATATTGA
NSGT1_Chm   1261 CAAGTTAATTTGAAGGCAATGGAATTGAGTGAAAAGATAAAATTGAAAGCTATTGATGAAAGGGTTAAAAAGTTATTGAAGCTTTTGTGTTGA
```

Supplemental Figure S1. DNA alignment of *S. lycopersicum NSGT1* (GenBank: KC696865.1) CDS with the consensus sequence of this gene extracted from Illumina Solexa sequencing data of *S. chmielewskii*. The full set of Illumina Solexa reads of S. chmielewskii was pre-processed and mapped onto the S. lycopersicum NSGT1 sequence reference using the CLC Genomic Workbench software (http://www.clcbio.com/products/clc-genomics-workbench/).
