# Supplementary material for: Identification of Loci Affecting Accumulation of Secondary Metabolites in Tomato Fruit of a Solanum lycopersicum × Solanum chmielewskii Introgression Line Population
Source: Front Plant Sci. 2016 Sep 28;7:1428. doi: 10.3389/fpls.2016.01428 (PMC5040107; doi:10.3389/fpls.2016.01428)
Supplement: Supplementary file 11 [file Image_2.PDF]

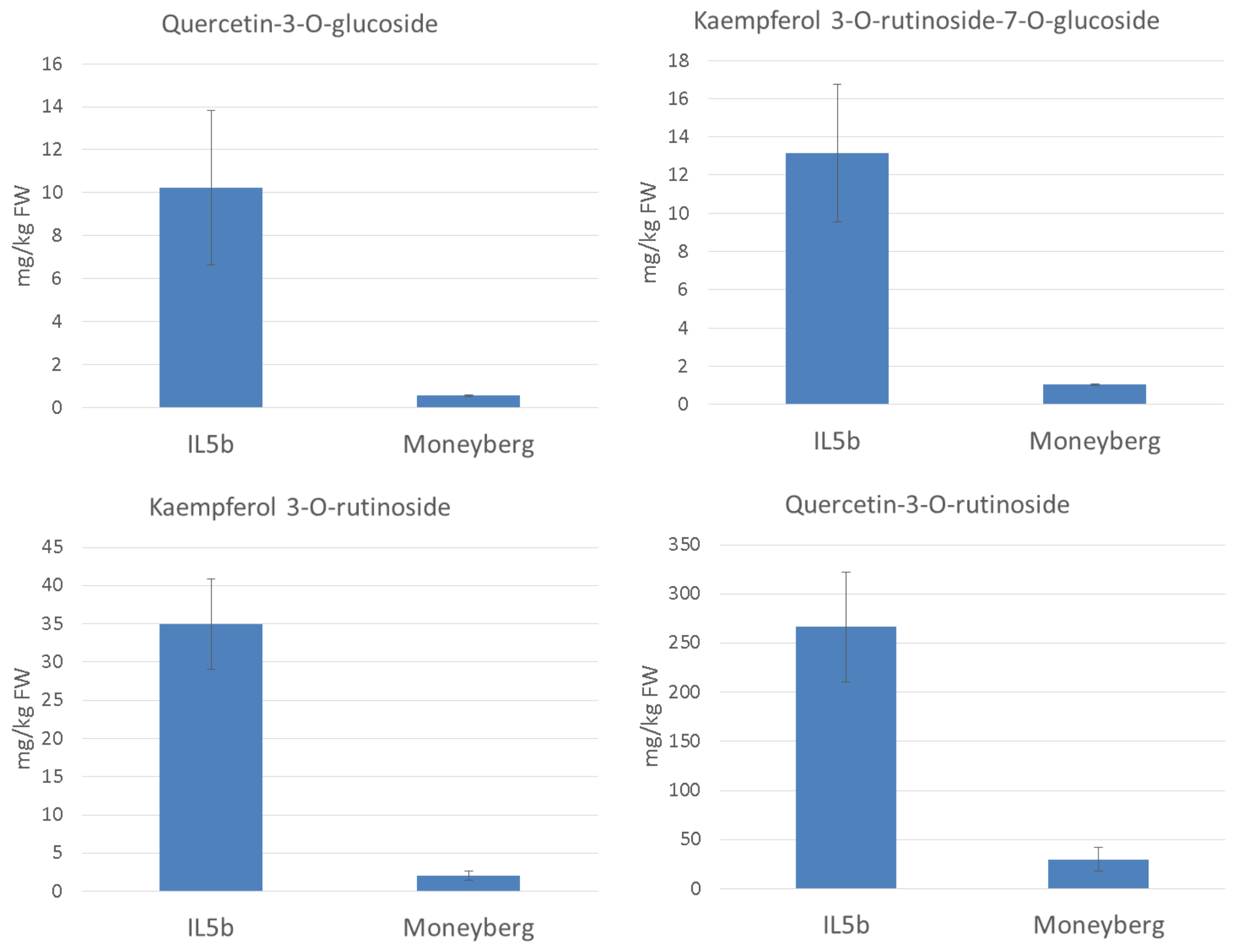

Supplemental Figure S2. Quantitative difference in accumulation of flavonols in fruit peel of IL5b compared to fruit peel of cv. Moneyberg
