# Supplementary material for: Identification of Loci Affecting Accumulation of Secondary Metabolites in Tomato Fruit of a Solanum lycopersicum × Solanum chmielewskii Introgression Line Population
Source: Front Plant Sci. 2016 Sep 28;7:1428. doi: 10.3389/fpls.2016.01428 (PMC5040107; doi:10.3389/fpls.2016.01428)
Supplement: Supplementary file 12 [file Image_3.PDF]

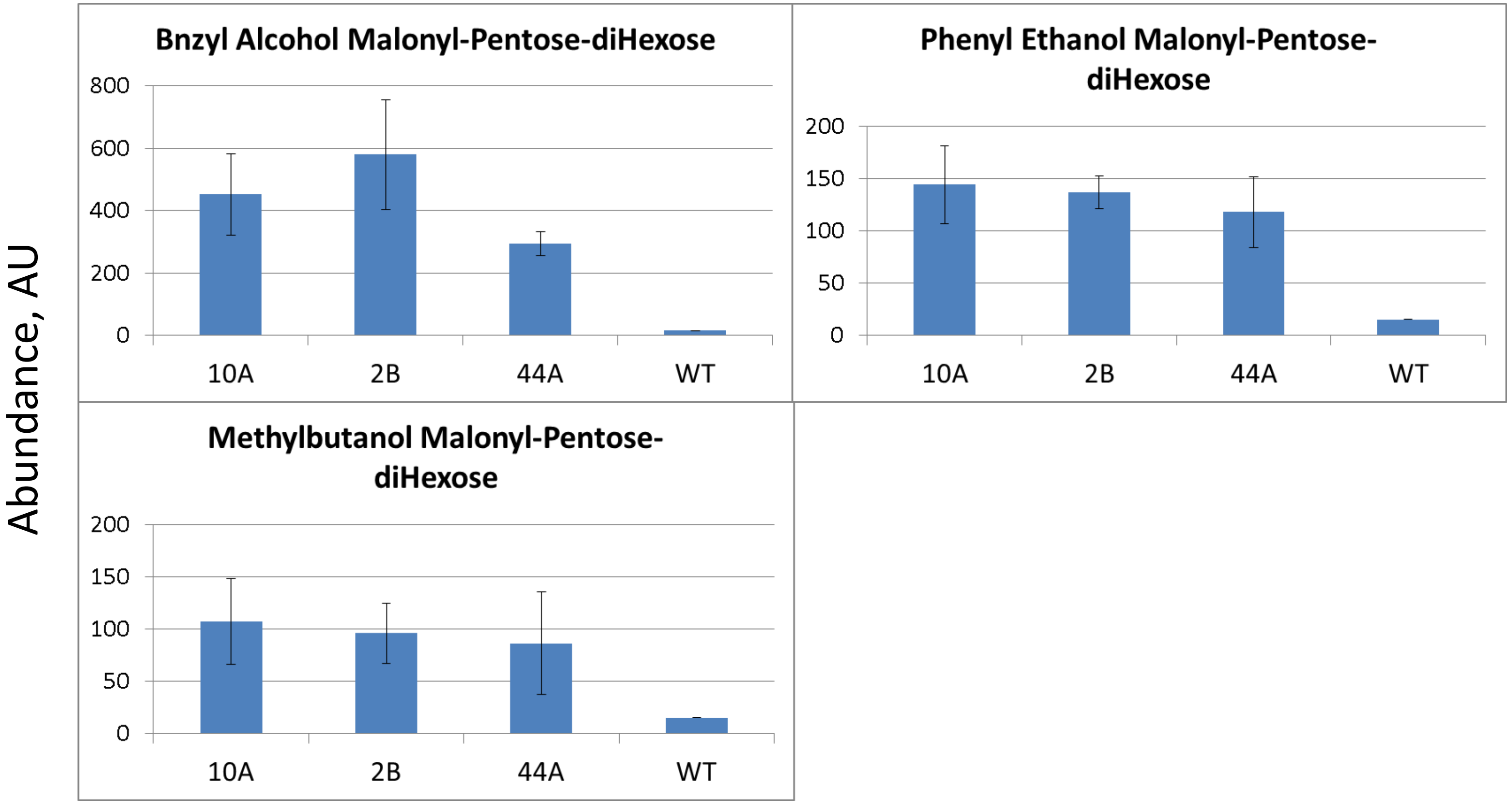

Supplemental Figure S3. Triglycosides of benzyl alcohol, methylbutanol and phenyl ethanol in fruits of independent transgenic cv. Moneymaker plants overexpressing *NSGT1* (10A, 2B and 44A) compared to the non-transformed control (WT).
