# Supplementary material for: Identification of Loci Affecting Accumulation of Secondary Metabolites in Tomato Fruit of a Solanum lycopersicum × Solanum chmielewskii Introgression Line Population
Source: Front Plant Sci. 2016 Sep 28;7:1428. doi: 10.3389/fpls.2016.01428 (PMC5040107; doi:10.3389/fpls.2016.01428)
Supplement: Supplementary file 13 [file Image_4.PDF]

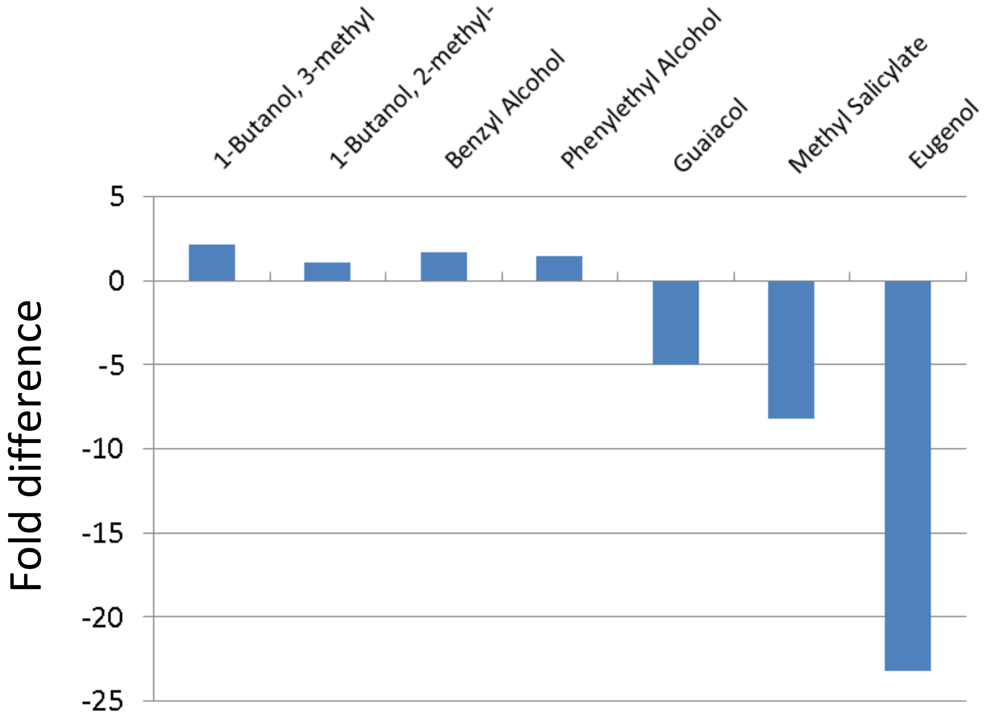

Supplemental Figure S4. The fold difference in volatile release from fruits of IL9d compared to fruits of cv. Moneyberg. The analysis of volatile compounds was performed on ripe fruit material of IL9d and cv. Moneyberg using a headspace GC-MS analysis as described in Tikunov et al., (2013)
